# In Rural Eastern Ethiopia Hearing Loss Is the Most Frequent Disability during Childhood: A Community Based Survey

**DOI:** 10.1371/journal.pone.0152791

**Published:** 2016-05-05

**Authors:** Biftu Geda, Yemane Berhane, Nega Assefa, Alemayehu Worku

**Affiliations:** 1 School of Nursing and Midwifery, College of Health and Medical Sciences, Haramaya University, Harar, Ethiopia; 2 Addis Continental Institute of Public Health, Addis Ababa, Ethiopia; 3 School of Public Health, Addis Ababa University, Addis Ababa, Ethiopia; TNO, NETHERLANDS

## Abstract

**Background:**

The type and extent of childhood disability in Ethiopia is unknown due to lack of accurate and reliable data. This study tried to assess the magnitude and types of disabilities among children 0–14 years of age in eastern Ethiopia.

**Methods:**

We conducted a cross-sectional community-based study among households that are under demographic and health surveillance in eastern Ethiopia. The study population consisted of all children aged 0–14 year. A structured questionnaire was used to assess the type and severity of the disability.

**Results:**

A total of 21,572 children in the age group 0–14 were screened for disability. Of which 586 (2.7%; 95% CI = 2.5%, 2.9%) had at least one kind of disability at the time of the survey. The proportion of disability increased as children were older; measured by the extended Mantel-Haenszel (M-H) chi square for linear trend (M-H = 48.74; P<0.001). Hearing impairment was the most common reported disability; 417 (71.2%; 95% CI = 67.5%, 74.9%). Among children with a disability, 179 (31.0%; 95% CI = 27.3%, 34.7%) had a combination of multiple disabilities and about a third, 200 (34.1%; 95% CI = 30.3%, 37.9%) had developed the disability during infancy. Magnitude of disability was higher among boys 335 (2.98%; 95% CIs = 2.66%, 3.30%) compared to girls 251 (2.44%; 95% CIs = 2.14%, 2.74%).

**Conclusion:**

Childhood disability is a health challenge in the study area and is already common at an early age. Permanent disability among children may be prevented by an early screening program in the routine child health services and adequate care, especially for hearing impairment.

## Introduction

Childhood disability is the gap between a child’s functional abilities and the demands of their social and physical environments [1, 2]. The disability definition for children is focused on their ability to play and later on their school performance [2]. Children with disabilities may suffer from long-term physical, mental, intellectual or sensory impairments that may hinder their full and effective participation in society on an equal basis compared to those without a disability [1, 3].

Although disability is believed to be prevalent among children in Africa, official statistics are unavailable either due to lack of reliable data or due to an attempt to conceal the extent of the problem [4]. Thus, the vast majority of children with disabilities are not recognized by national policy makers [5] and thus completely cut off from health, education and other social services to which they should be entitled to [6].

Most countries in Africa depend on UN estimates, which reported about 5% of children in the age bracket of 0–14 years as having one or more disability [6]. Other African countries estimate the extent of disability through either analysis of secondary data from small surveys [7–9] or by adding disability related questions to a population census [10] without following the recommended data collection methods for disability survey [4]. The purpose of this study was therefore to assess the prevalence, types and severity of disability among 0–14 year old children in Eastern Ethiopia. The study focused on this age group to show the importance of early detection and early intervention[4, 9, 11, 12] and also ensures a planned transition to independent adulthood [5].

## Methods and Procedures

This study was conducted from January to April 2014 in Kersa Health and Demographic Surveillance System (Kersa HDSS), which is located in Kersa District, Oromia Region, in the eastern Ethiopia. The district, also known as *woreda* in Ethiopia, has a total number of 38 *Kebeles* (the lowest administrative units). Kersa HDSS has been operational in 12 of the kebeles since June 2007. The surveillance covers about 13,000 households (HHs) with a total population of 63,000 at the time of the survey. All households are visited twice a year to update vital events [13]. Our survey, a cross-sectional community based study, included all households participating in the surveillance system that had eligible children (0–14 year old). No household with eligible children was excluded from the study. As per report of Kersa HDSS, the number of children aged 0–14 years in the study area was 25,200 [13].

A structured household questionnaire was adapted based on previous study tools [9, 14–18]. The contents of the questionnaire has been shown to be fairly reliable and valid for detecting moderate to severe forms of disability [14, 17, 19, 20]. In addition, the tool was easy to administer since it was designed to identify child’s physical challenges that were of great concern to immediate caregivers and that were easy to remember [15]. Screening questions were adopted from UNICEF ‘Ten questions (TQs)’ and Washington Group Short Set (WGSS) questions [16, 21]. The tools have been used in several low income countries, where post screening verification by specialist is not possible due to resource constraints. It is also simple and inexpensive to screen large number of children at a time [14, 15, 17]. After pilot testing, we added two questions, including the mental health problem identified as a concern by both the community and research team. The second one is a question for probing which was already in TQs questions (naming object) but we treated it separately in order not to lose vital information. Therefore, TQs questions become 10+2 questions in this particular study.

The English questionnaire was translated to the local language (*Afan Oromo*) by language experts who are competent in both English and *Afan Oromo*. It was then piloted in a similar setting before being used in the actual survey. As noted previously, following pilot testing, we increased the number of questions from 10 to 12. Data were collected by lay interviewers who had at least 10 years of education and who received a three day training given by the researchers. In addition, data collectors were accompanied by a person with a disability during the data collection in order to increase acceptance. The primary respondents were the biological mothers of the eligible children. In the event the biological mother was not available, either the biological father or other adult guardians were interviewed. Regular supervision was done by experienced field research supervisors and the investigators.

The questionnaire was administered to the caregiver of eligible children in two steps. First all study participants were interviewed using the disability screening questionnaire. Then, details about the disability were inquired only from parents who reported having a child with at least one kind of disability. We studied 12 domains of disabilities, which include vision, hearing, sitting and standing, mobility, seizure, understanding others (listening and communicating), to be understood by others (speaking words that have meaning and which others can understand), speech, learning, object naming, mental retardation and mental health.

The response levels of all the 12 domains were four (no disability, some disability, a lot of disability and cannot do at all) as per suggestions of different studies [16, 22, 23]. A child was considered to have disability if he/she has at least ‘some disability’ on any one of the 12 disability domains [9, 20]. To determine childhood disability (CHD) magnitude, we followed the following steps: 1. we created a single variable of ranked composite score from the 12 disability domains. 2. the outcome variable of ranked response was then transformed to the outcome variable of a binary category where YES = 1 (child with some disability, a lot of disability and can’t do at all), and NO = 0 (Normal child). Then, children were classified into either having disability (yes) or not (no) group; where yes = 1 (child with at least ‘some disability; no = 0 (child with no disability).

The disability status was further stratified by age, sex, type of disability, severity of disability depending on the caregiver report, number of disabilities and household wealth index. The household wealth index was developed based on nine asset variables using principal component analysis [23–26]. Then, the score was divided into five quintile categories (0 = Lowest, 1 = Low, 2 = Middle, 3 = High, 4 = Highest). In addition, we constructed the following three composite indices based on a theoretical model of a multi-dimensional concept [16, 27]. These are learning disability from learning problem, mental retardation, difficulty related to naming objects); motor disability from problems related to extremities, sitting and standing; and communication disability from problems related to speech, understanding others and to be understood by others. Then, they were dichotomized for descriptive analysis and we used all children of the population aged 0–14 years old as a denominator for better comparisons.

Each completed questionnaire was checked for completeness and consistency at the end of each data collection day. Data were entered and cleaned using EPIDATA version 3.1. Then, cleaned data were exported to Stata version 12 the statistical software used for our analysis. Descriptive statistics were used to calculate the frequency distribution, proportions with 95% confidence interval (categorical variables), and mean and standard deviation (continuous variables). To assess whether group differences were statistically significant, we used the Pearson chi square test and the extended Mantel-Haenszel (M-H) chi square test for linear trend, p-value set at 0.05. For the extended Mantel-Haenszel (M-H) chi square test for linear trend, we used openEpi-Dose response Chi square for Trend version-3 [28]. Data for open epi were first summarized using two by two tables and then the summarized data were entered in to open epi for analysis.

Ethical approval was obtained from the Institutional Review Board (IRB) of Haramaya University and from the National Research Ethics Review Committee at the Ministry of Science and Technology. We also obtained permission to conduct the study from the local administrative offices. Participation into the study was entirely on a voluntary basis. Participants received comprehensive information about the study and were assured of confidentially and protection of their privacy before giving consent. We referred treatable disabilities to the nearby health facilities and facilitated linking children to specialized services and concerned agencies available in the area. We took written consent from proxy respondents because in Ethiopia, for the children aged 0–14 years, parents are responsible for the consent. We read the consent for them because of illiteracy and if they agree they sign before the interview. An information leaflet is attached to the first part of the questionnaire and then a signature was kept with each questionnaire. All the procedures were approved by the ethics committees/IRBs of Haramaya University and the Ministry of Science and Technology.

## Result

A total of 25,200 households with eligible children were invited for the survey of which 21,572 agreed to participate with a response rate of 86.0%. For 20,846 (96.6%) of the children, respondents were biological mothers, 318 (1.5%) were biological fathers, and the remaining 408 (1.9%) were non-biological guardians.

The mean and standard deviation (SD) of the children’s age at the time of the survey was 6±4 years. Almost all children, 20,444 (94.8%), were born at home and 20,798 (96.4%) of the study children were living with both parents. Only 2,508 (11.6%) of children’s mothers were literate while 6,577 (31.5%) of the children’s fathers were literate (Table 1).

**Table 1:** General characteristics of respondents and index child, Kersa District Eastern Ethiopia, 2014.

Variables	n	%
**Child current age (n = 21,516)**		
<2	4,221	20.0
2–4	4,544	21.0
5–9	8,428	39.0
10–14	4,323	20.0
**Sex (21,527)**		
Female	10,274	52.3
Male	11,253	47,7
**Child birth order (n = 21,505)**		
2^nd^ and below	8,642	40.0
Third	3,826	18.0
Fourth	3,142	25.0
5^th^ and above	5,895	27.0
**Child birth place (n = 21,525)**		
Home	20,403	95.0
Health clinic	1,122	5.0
**Child mode of delivery (n = 21,422)**		
Spontaneous vaginal delivery	21,316	99.5
Cesarean section	106	0.5
Parental status (n = 21,525)		
Two parents	20,756	96.0
One parent	769	4.0
**Mother’s age during child birth (n = 21,527)**		
15–19	2,026	9.0
20–24	7,020	33.0
25–29	6,563	30.0
30–34	4,006	19.0
35+	1,912	9.0
**Mother’s education (n = 21,507)**		
Illiterate	19,044	88.0
Literate	2,508	12.0
**Father’s education (n = 21,477)**		
Illiterate	14,911	69.0
Literate	6,566	31.0
**Household socioeconomic index (n = 21,431)**		
Lowest	4,299	20.0
Low	4,348	20.0
Middle	4,247	20.0
High	4,658	22.0
Highest	3,879	18.0
**Overall disability status (n = 21,572)**		
Children without disability	20,986	97.3
Children with disability	586	2.6
**Disability status of children aged 2–9 years old (n = 12,991)**		
Children without disability	12,649	97.4
Children with disability	342	2.6

**Fig 1:**
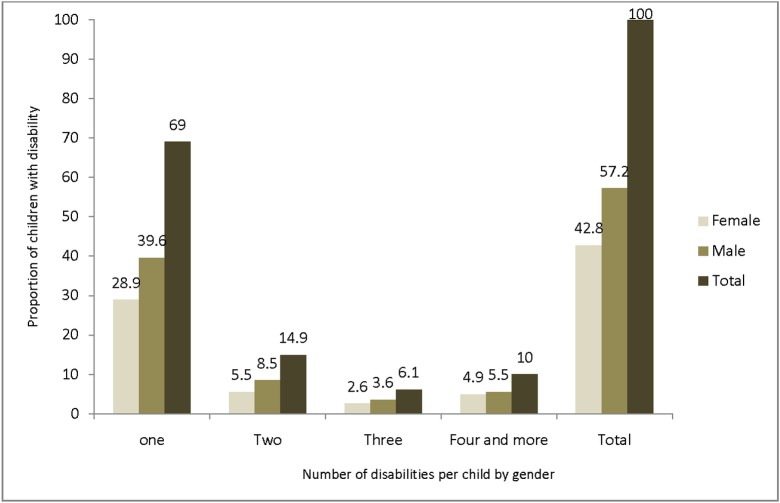
Number of disabilities per child by sex in Kersa district Eastern Ethiopia, 2014.

**Fig 2:**
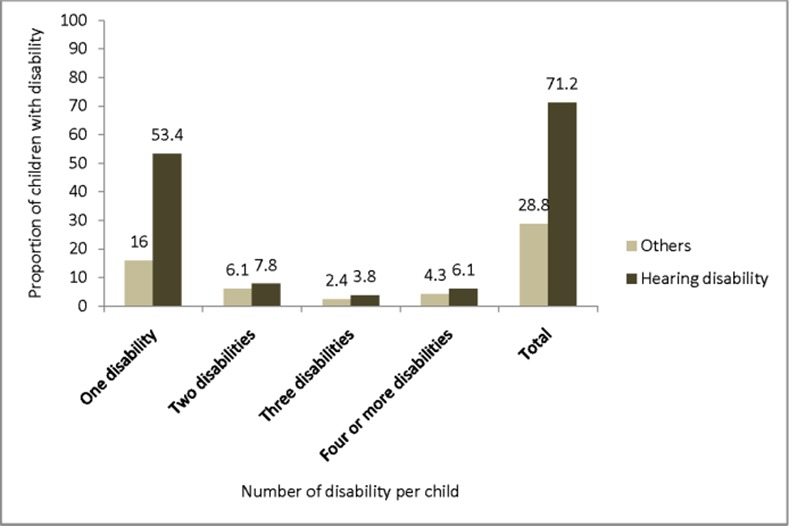
Proportion of hearing impairment among other disabilities in Kersa district Eastern Ethiopia, 2014.

Overall, 586 (2.7%; 95% CI = 2.5%, 2.9%) of the screened children aged (0–14) had a disability (Table 1). Among the children with a disability, 132 (0.61%; 95% CI = 0.50%, 0.71%) had severe to very severe forms of disability (Table 2). One hundred and seventy nine (31.0%) of the children had multiple disabilities (Fig 1). On average, the numbers of disabilities per child was about two and the contribution of hearing impairment is high 417 (71.2%; 95% CI = 67.5%, 74.9%) in children with multiple disabilities (Fig 2).

**Table 2:** Prevalence of disability by degree of severity and according sex in children aged 0–14 year, living in rural eastern Ethiopia (Kersa District), 2014.

Disability category	Degree of severity by sex								Total category prevalence by sex					
	Mild to moderate				Severe to very severe									
	boys		girls		boys		girls		boys		girls		both sexes	
	n	%	n	%	n	%	n	%	n	%	n	%	n	%
Vision	28	0.25	29	0.28	16	0.14	8	0.08	44	0.39	37	0.36	81	0.38
Hearing	169	1.50	133	1.30	72	0.63	43	0.42	241	2.14	176	1.71	417	1.94
Sitting and standing	19	0.17	16	0.16	14	0.12	13	0.13	33	0.29	29	0.28	62	0.29
Mobility	19	0.17	12	0.12	13	0.12	11	0.11	32	0.28	23	0.22	55	0.26
Seizure	6	0.06	4	0.04	11	0.10	12	0.12	17	0.15	16	0.16	33	0.15
Understanding others	24	0.21	20	0.19	16	0.14	10	0.10	40	0.36	30	0.29	70	0.33
Understood by others	21	0.19	16	0.16	13	0.12	13	0.13	34	0.30	29	0.28	63	0.29
Speech	14	0.12	18	0.18	16	0.14	9	0.09	30	0.27	27	0.26	57	0.26
Learning	20	0.18	15	0.15	16	0.14	13	0.13	36	0.32	28	0.27	64	0.30
Naming object	10	0.09	5	0.05	14	0.12	9	0.09	24	0.21	14	0.14	38	0.18
Mental retardation	15	0.13	10	0.10	15	0.13	8	0.08	30	0.27	18	0.18	48	0.22
Mental health	16	0.14	12	0.12	19	0.17	9	0.09	35	0.31	21	0.20	56	0.26
Child with at least one disability	256	2.27	198	1.93	79	0.70	53	0.52	335	2.98	251	2.44	586*	2.72

The total children screened were 21,527 of which 10,274 were girls and 11,253 boys. Prevalences were calculated based on these numbers.

* The sum of the categories is greater than the children with disabilities observed (586) because of multiple disabilities reported for some of the children. One hundred seventy nine (31.0%) children studied had two or more disabilities. Therefore, the total row reported is the number of children with the respective disability observed.

**Fig 3:**
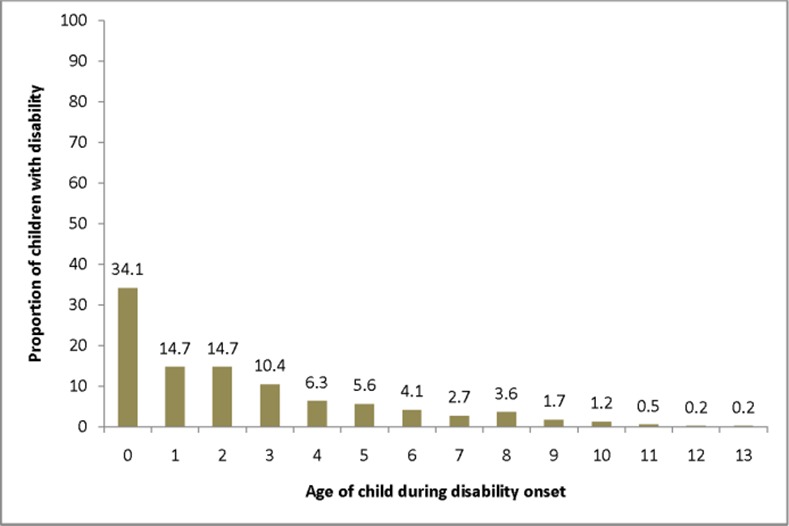
Distribution of children with a disability by the age of disability onset in Kersa district Eastern Ethiopia, 2014.

The prevalence of disability increases as a child’s age increases, which is statistically significant using the extended Mantel-Haenszel (M-H) chi square for linear trend (M-H = 48.74; P<0.001). Prevalence of each age category were 68 (0.32%; 95% CIs = 0.24%, 0.40%), 129 (0.60%; 95%CIs = 0.50%, 0.70%), 213 (0.99%; 0.86%, 1.12%) and 176 (0.82%; 95% CIs = 0.70%, 0.94%) for age group <2, 2–4, 5–9 and 10–14 respectively (Table 3). The mean age and SD for the disability onset was 2.5±2.7 years. For 200 (34.1%; 95% CI = 30.2%, 37.8%) of the children with a disability, the onset of the disability was during infancy (Fig 3).

**Table 3:** Disability classification by age, in children aged 0–14 year living in rural eastern Ethiopia (Kersa District), 2014.

Disability category	Age disaggregated prevalence								Overall prevalence (n = 21,572)		95% CIs**
	<2(n = 4,247)		2-4(n = 4,542)		5–9 (n = 8,449)		10-14(n = 4,334)		n	%	
Vision (n = 81)	3	0.07%	12	0.26%	38	0.45%	28	0.65%	81	0.38	0.30%, 0.46%
Hearing (n = 417)	57	1.34%	93	2.05%	137	1.62%	130	3.0%	417	1.94	1.76%, 2.12%
Sitting and standing(62)	6	0.14%	22	0.48%	20	0.24%	14	0.32%	62	0.29	0.22%, 0.36%
Mobility (n = 55)	4	0.09%	18	0.40%	20	0.24%	13	0.30%	55	0.26	0.19%, 0.33%
Seizure (n = 33)	2	0.05%	5	0.11%	19	0.22%	7	0.16%	33	0.15	0.10%, 0.20%
Understanding others(70)	2	0.05%	13	0.29%	30	0.36%	25	0.58%	70	0.33	0.25%, 0.41%
Understood by others(63)	1	0.03%	8	0.18%	29	0.34%	25	0.58%	63	0.29	0.22%, 0.36%
Speech (n = 57)	2	0.05%	14	0.31%	22	0.26%	19	0.44%	57	0.26	0.19%, 0.33%
Learning (n = 64)	2	0.05%	12	0.26%	26	0.31%	24	37.6%	64	0.30	0.23%, 0.37%
Naming object (n = 38)	2	0.05%	11	0.24%	12	0.14%	13	0.30%	38	0.18	0.12%, 0.24%
Mental retardation(n = 48)	2	0.05%	5	0.11%	22	0.26%	19	0.55%	48	0.22	0.16%, 0.28%
Mental health (n = 56)	7	0.16%	13	0.29%	23	0.27%	13	0.30%	56	0.26	0.19%, 0.33%
Child with at least any one disability	68	0.32%	129	0.60%	213	0.99%	176	0.82%	586*	2.72	2.50%, 2.94%

** CIs = confidence intervals

* The sum of the categories is greater than the children with disabilities observed (586) because of multiple disabilities reported for some of the children. One hundred seventy nine (31.0%) of the children studied had two or more disabilities. Therefore, the total row reported is th number of children with the respective disability observed.

The magnitude of disability was higher among boys 335 (2.98%; 95% CI = 2.75%, 3.21%) than in girls 251 (2.44%; 95% CI = 2.23%, 2.65%); (Pearson chi2 [1] = 5.24; P<0.05). The prevalence of disability of all categories was higher among boys (Table 2 & Fig 1). Two hundred and fifty six (2.27%; 95% CIs = 2.0%, 2.55%) of the boys and 198 (1.93%; 95% CIs = 1.66%, 2.20%) of the girls had a mild to moderate forms of disability whereas 79 boys (0.70%; 95% CIs = 0.55%, 0.90%) and 53 girls (0.52%; 95% = 0.38%, 0.66%) had a severe to very severe forms of disability (Table 2). The prevalence calculated per 100,000 children tends to be higher among boys 335 (2977; 95% CIs = 2.66%, 3.30%) as compared to girls 251 (2443; 95% CIs = 2.14%, 2.74%) though this is not significant (Table 2).

Prevalence of childhood disability (CHD) was high among households with low socioeconomic status (M-H = 28.44; P<0.001). Generally, the proportion of children with one or more disabilities was also higher in the lowest wealth quintile (Pearson chi2 (6) = 29.31; p<0.001).

**Fig 4:**
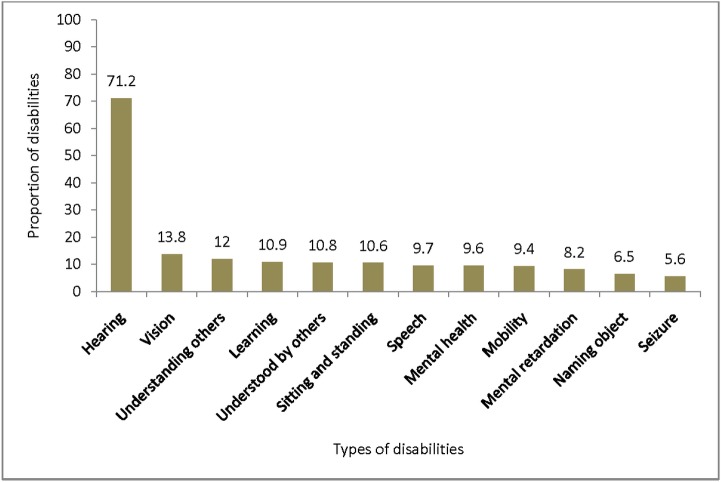
Proportion of specific disabilities by type in Kersa district Eastern Ethiopia, 2014.

Hearing impairment was the most common type of disability reported 417(1.93%; 95% CI = 1.75%, 2.11%). Of the children with a hearing disability, 171 (41.0%; 95% CI = 36.3%, 45.7%) had chronic ear discharge (Pearson chi2 (3) = 35.51; p<0.001). Communication problems found in 105 children (0.49%; 95% CIs = 0.40%, 0.58%); learning problems in 89 (0.41%; 95% CIs = 0.32%, 0.50%); Visual impairment in 81(0.38%; 95% CI = 0.30%, 0.46%) and hampered mobility was found in 79 children (0.37%; 95% CI = 0.29%, 0.45%): these were the four most common reported disabilities following hearing impairment. Seizures were reported in 33 children (0.15%; 95% CI = 0.10%, 0.20%) (Table 2 & Fig 4).

## Discussion

This survey identified the magnitude and types of disabilities among children aged 0–14 year old in Eastern Ethiopia using a recommended approach and validated assessment tool. Although prior studies in Ethiopia have tried to document the magnitude and types of disability among the general population [7, 8, 29], they did not comprehensively address all categories of disability [6, 10, 29, 30] and some did not stratify the disability by age [1]. Therefore, our study is the first to report data in detail on childhood disabilities in rural eastern Ethiopia.

In our study, childhood disability has an early onset and hearing impairment is the most prevalent form of disability. In addition, a large proportion of the disabilities resulted in multiple handicaps and all types of disabilities were more common among boys. Hearing impairment was associated with chronic ear infections and the majority of children had a potentially treatable hearing problem. Disability in this context may be attributed to poor access to health care and neonatal health services, lack of awareness about possible causes of disabilities, and folk healers [4, 6, 9]. In addition, poverty [23, 26], fear of stigma [31], and lack of attention from health professionals [4, 5, 32] may also be associated with childhood disabilities. Communication, learning, vision and hampered mobility were common problems in this study and these findings are consistent with findings reported in other middle and low income counties [9, 15, 18, 23]. However, direct and detailed comparisons could not be made due to variation in age groups studied, and definitions used for forms of disabilities, and study settings.

In this study, prevalence of disability increases as a child’s age increases and this finding is consistent with other studies [9]. On the other hand, prevalences of severe to very severe forms of disabilities are higher than in the World Health Organization’s report on disability of South East Asia and lower than the report on Africa [33] which could be attributed to differences in response categories [9, 34] and census based report [4, 35–37] which did not follow standard data collection methods [4].

The prevalence of childhood disability reported in our study is lower than the UNICEF estimate for our region, which is 5% and quoted widely since 2004 [6]. The estimate was based on few surveys that used inconsistent methods [6, 9, 31]. Other African studies have also produced disability prevalence estimates among children; their results ranged from 1.8% [31] to 16% [15].

Several factors hinder measuring disability prevalences accurately using a community survey. Low survival of children with disability (CwD) [4, 31, 38–40], failure to recognize and count those confined in institutions [6, 31, 39], those children hidden by families, or abended, who then live on the streets [2, 38]. This could lead to under estimation of the prevalence. Improved child survival due to advanced medical care, public health improvements, can also influence its prevalence [2, 12, 23, 39]. Our study could also be influenced by all these factors and the prevalence reported in this paper may be an underestimation of the actual magnitude of the problem.

Developmental disability of all forms can occur due to birth trauma like asphyxia [41–43]. Our study was conducted in an area where the majority of births are at home without proper medical provision. This may explain in part the high proportion of disabilities reported starting during the first year of life. Home delivery without proper medical supervision is a risk factor for early childhood disability [9].

In this study, hearing impairment was the most commonly observed disability; this is consistent with previous reports [9]. In developing countries 6 in 1,000 live births are either born with a form of hearing impairment or develop this during the neonatal period [42, 43]. Such high prevalence of hearing disability could be due to chronic ear infections common in Ethiopia [6, 39, 44]. A hearing disability developed during early childhood is likely to result in irreversible deficits in speech, linguistic, cognitive and educational development [45, 46]. Early detection and preventive measures coupled with timely and effective case management may prevent these [6, 44].

Disability was common among households with a lower socioeconomic status, which is consistent with other studies both in developing and developed countries [9, 39, 47]. However, the direction of association is being debated and whether poverty is causing disability or disability causing poverty needs further investigation [9, 39, 47].

The following is a serious limitation of our study. No physical examination was done by us and disability classifications were entirely dependent on the assessments of our responders. The use of proxy-reporters is likely to underestimate the prevalence of disability, due to reporting biases. This also could cause difficulties in categorizing the severity status [32]. Our study was conducted in those villages where residents have been subject to continuous data collection, which may negatively affect their response because of fatigue and lack of incentives. An overall 14% non-response may also indicate the taboo and stigma associated with disability in our communities.

We tried to minimize these biases by selecting biological mothers as the main source of information, by providing adequate training for our interviewers, by setting a convenient time for the interviews and by providing full privacy to our responders. We hope this would prevent elicit unbiased information as much as possible, also by using a standard data collection instrument which was validated in different other countries. In addition, we pre-tested our tool and piloted the full study procedure before conducting our main study. Data collectors were also supported by a person with a disability during the data collection in order to increase acceptance.

In conclusion, we find a relatively high proportion of children with disability in rural eastern Ethiopia. Hearing impairment was the major disability with an onset during infancy and associated with chronic ear infection.
